# T2 relaxometry in the extremely-preterm brain at adolescence

**DOI:** 10.1016/j.mri.2015.12.020

**Published:** 2016-05

**Authors:** Nicholas Dingwall, Alan Chalk, Teresa I. Martin, Catherine J. Scott, Carla Semedo, Quan Le, Eliza Orasanu, Jorge M. Cardoso, Andrew Melbourne, Neil Marlow, Sebastien Ourselin

**Affiliations:** a Department of Computer Science, University College London, UK; b Centre for Medical Image Computing (CMIC), University College London, UK; c Academic Neonatology, EGA UCL Institute for Women's Health, London, UK

**Keywords:** Prematurity, T2 relaxometry, Myelin, White matter

## Abstract

Survival following very preterm birth is associated with cognitive and behavioral sequelae, which may have identifiable neural correlates. Many survivors of modern neonatal care in the 1990s are now young adults and the evolution of MRI findings into adult life has rarely been evaluated. We have investigated a cohort of 19-year-old adolescents without severe impairments born between 22 and 26 weeks of gestation in 1995 (extremely preterm: EP). Using T2 data derived from magnetic resonance imaging we investigate differences between the brains of 46 EP participants (n = 46) and the brains of a group of term-born controls (n = 20). Despite EP adolescents having significantly reduced gray and white matter volumes, the composition of these tissues, assessed by both single and multi-component relaxometry, appears to be unrelated to either preterm status or gender. This may represent either insensitivity of the imaging technique or reflect that there are only subtle differences between EP subjects and their term-born peers.

## 1 Introduction

Globally the major causes of neonatal death comprise complications related to preterm birth [8]. In the developed world survival has now improved at all gestational ages and particularly for those born at extremely low gestations (22–26 weeks; extremely preterm; EP). However EP births are at greatly increased risk of long-term morbidities, such as learning difficulties or cerebral palsy, and prevalence of these conditions has not significantly altered. Moore et al. [12] reported that, despite a rise in the number of neonatal admissions of 44%, survival of EP births rose by 15% between 1995 and 2006, but that the proportion with significant neurocognitive morbidity was unchanged. Thus there is a need for research into the causes and predictors of adverse outcome in this group, if for no other reason than shortening study times and avoiding the need to wait for many years of follow-up. Although differences on brain imaging findings associated with impairment are important it is also important to understand the eventual outcomes after remodeling over childhood and adolescence. Such data could inform the identification of key areas for investigation at younger ages.

The brain develops rapidly during the period coinciding with ex-uterine development after EP birth. In particular myelination may be disturbed during this period, potentially because of injuries to immature oligodendroglia as a result of inflammation or hypoxia [17] and provides one potential biomarker of interest [13], [14]. Myelin surrounds the neuronal axons and increases the efficiency and speed of conduction of signals, and thus disturbances in its development may be linked to brain function. Myelination commences at around 28 weeks of gestation and continues into infancy [1], [4], [11] coinciding with the period of maximal physiological instability in the early weeks after birth at EP gestations.

Magnetic resonance (MR) T2 relaxometry is a non-invasive imaging technique that may be used to measure the myelin water fraction within the brain [9]. The decay of the signal in a T2 weighted sequence follows an exponential relationship related to the spin density and the native T2 of the tissue. By acquiring images at different echo times this equation can be fitted for a given voxel and the time constant T2 can be found. The measured T2 of a tissue is dependent upon how it binds water and therefore T2 values vary with tissue type. Importantly, the water trapped within myelin sheaths is very tightly bound and therefore has a very short T2 compared to other tissues. Therefore, the T2 of a voxel can be used to infer tissue composition, and in particular to determine the fraction of myelin water across the brain. We have reported differences in the white matter T2 values between the brains of preterm babies and those of full term babies [5]. These values were independent of visually identified changes, termed diffuse and excessive high signal intensity, thought previously to be a biomarker [3] Importantly T2 values were correlated with developmental scores at 2 years of age [5]. However these averaged data may not reflect structures because most brain tissue consists of multiple tissue types with distinct T2 values. Therefore it is better represented by a multi-compartment model of multi-exponential decays, which has been explored by number of authors [9], [10], [15] to extract a specific estimate of the myelin water fraction and a long-T2 component associated with free-water spaces such as cerebrospinal fluid (CSF).

Previous work has established some correlation between estimated myelin water fraction and myelin: Laule et al. [6], [7] validated the myelin water (MW) fraction as a surrogate measure for myelin density. MacKay et al. [9] also support the strong correlation between MW fraction and myelin. We focus on three tissue types: MW being part of white matter (WM), intra- and extra-cellular water being part of both white and gray matter (GM), and CSF. Hence, multi-echo T2 relaxometry may be used to investigate both myelination and the quantity and location of different tissue types in the brain [16]. If we can determine the spatial distribution of myelin density from T2 relaxometry, this will allow an in vivo comparison of myelin distribution and density between subjects.

In this paper we investigate the differences in MR visible myelin in 46 19-year-old EP subjects and 20 control individuals born at full term. We hypothesize that accurate measurement of myelin density in the brains of preterm born adolescents may help to predict cognitive impairment, and that this cognitive impairment is related to a persistently reduced myelin density. Our work provides a comparison of the white matter myelin density between premature births and term births at adolescence and whether this can be used to indicate a long-term outcome of premature birth.

## 2 Materials and methods

### 2.1 Data

Data were collected at 19 years of age from 46 EP adolescents (mean gestation at birth 24 weeks and 6 days (range 22^+1^–25^+6^weeks)) and 20 term-born subjects (recruited as classmates in childhood at 6 or 11 years). Individuals were imaged using a Philips 3 T Achieva. We acquired T2 weighted data with ten echo times at TE = {13, 16, 20, 25, 30, 40, 50, 85, 100, 150}ms (2.5 × 2.5 × 3.0 mm). In addition we acquired a 3D T1-weighted volume at 1 mm isotropic resolution (TR/TE = 6.78/3.06 ms) to use for segmentation and region labels [2]. Three datasets were rejected, two in subjects with enlarged lateral ventricles and one with severe motion artifact, leaving 43 EP subjects (16 male (mean gestation 25^+0^w), 27 female (24^+7^w)) and 20 controls (10 male, 10 female).

### 2.2 T2 relaxometry

In single-component T2 relaxometry, we start by noting that the signal decays exponentially in most voxels, and therefore begin by considering models of the form

(1) $$S_{\mathrm{TE}}=S_{0}\;e^{-\mathrm{TE}/T2}\;$$

We can estimate a fixed iteration solution by linearizing this equation and using a linear least squares routine to estimate the two parameters S0 and T2.

(2) $$\ln S_{\mathrm{TE}}=\left[1,\mathrm{TE}\right]\left[{}_{-1/T2}^{\ln S_{0}}\right]$$

The results of the linear fitting are used to initialise a non-linear least squares fit, which maintains the original noise distribution of the data. We minimize gradients of the original function (Eq. (1)).

(3,4) $$\begin{array}{l} \frac{\delta S_{\mathrm{TE}}}{\delta S_{0}}=e^{-\mathrm{TE}/T2}\\ \frac{\delta S_{\mathrm{TE}}}{\delta T2}=S_{0}\frac{\mathrm{TE}}{{T2}^{2}}\;e^{-\mathrm{TE}/T2} \end{array}$$

The parameter estimates can then be updated using the following non-linear update step until convergence is reached; either the model fit residual does not change significantly (∆ r < 1e − 6) or the maximum number of iterations is reached (500).

(5) $$\left[{}_{{T2}^{i+1}}^{S_{0}^{i+1}}\right]=\left[{}_{{T2}^{i}}^{S_{0}^{i}}\right]+{\left(D^{T}\;D\right)}^{-1}\;D^{T}\;\left(S_{\mathrm{TE}}-S_{0}^{i}\;e^{-{\mathrm{TE}/T2}^{i}}\right)$$

(6) $$D=\left[\frac{\delta S_{\mathrm{TE}}}{\delta S_{0}^{i}},\frac{\delta S_{\mathrm{TE}}}{\delta {T2}^{i}}\right]\;$$

### 2.3 Multi-component T2 relaxometry

One limitation of the single component model is that it considers each voxel as a single homogeneous piece of matter, a significant simplification of the biology [9]. An important advantage of the multi-compartment model is that it allows us to estimate the fractions of different kinds of matter in a voxel, which we will need to do to estimate the amount of myelin in a brain. We know that the brain actually consists of several different types of matter, each with its own relaxation time, and so we also consider multi-compartment models of the form:

(7) $$\begin{array}{l} S\left(\mathrm{TE},\left\{T2\right\}\right)=S_{0}\sum_{i}v_{i}e^{-\mathrm{TE}/T2}\\ \text{subject}\;\mathrm{to}\;v_{i}>0\forall v_{i}\;\mathrm{and}\;\sum v_{i}=1_{\left(7,8\right)} \end{array}$$

where we seek the n volume fractions *v*_*i*_ which represent an exponential decay with a given T2_*i*_, similar to Raj et al. [16]. The restrictions that all *v*_*i*_ must be greater than or equal to zero and that the sum of all *v*_*i*_ must equal 1 apply. An effective approach to implement the first of these constraints is to use non-negative least squares. This minimizes a cost function that includes an L1-norm on parameters, and so encourages a sparse representation, which means that we can fit arbitrarily many compartments without simply reconstructing the data in parameter space. Previous work [9], [15] suggests T2 of 10–50 ms for myelin water, 70–90 ms for white and gray matter and greater than 2 s for CSF. The longest echo time in our dataset is 150 ms but in our model CSF can be accounted for by the longest available decay time. We fit a three-component model using literature values of expected T2s of [20, 80, 2000] ms and assign the result of the estimate of v^20ms^ to the myelin water fraction.

### 2.4 Image segmentation and parcellation

**Fig. 1:**
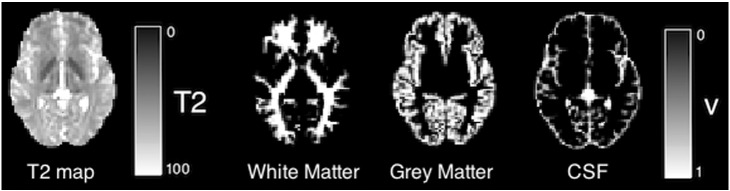
Example T2 map and corresponding three-class tissue type segmentation.

T1-weighted data are masked, segmented and labeled using the unified Geodesic Information Flow strategy of Cardoso et al. [2]. Segmentations are resampled into the space of the T2 weighted images and used to define regions of interest for total gray and white matter (see Fig. 1).

### 2.5 Data analysis

Data analysis is carried out using MATLAB 8.1 (Mathworks Inc., MA). We group the data by both gender and extreme-preterm status. We carry out standard Student's t-tests between gender and preterm groups for both T2 parametric maps and for brain tissue volume and report significance for a p-value of less than 0.05.

### 2.6 Markov-chain monte-Carlo myelin density estimation

To analyze the distribution of myelin density values we obtain, we carry out a Markov-chain Monte-Carlo (MCMC) analysis of the parameter space of the data. For each subject we find the average white matter signal intensity and use this as the data for the MCMC analysis, finding the average preterm white matter signal and average term-born white matter signal. The parameter distribution of compartment volume fractions is modeled from a Direchlet distribution. We use a burn in period of 100,000 iterations and run for a further 100,000 iterations. After thinning we keep 5000 samples to form our parameter distributions.

## 3 Results

### 3.1 Tissue volume in the preterm adolescent brain

**Fig. 2:**
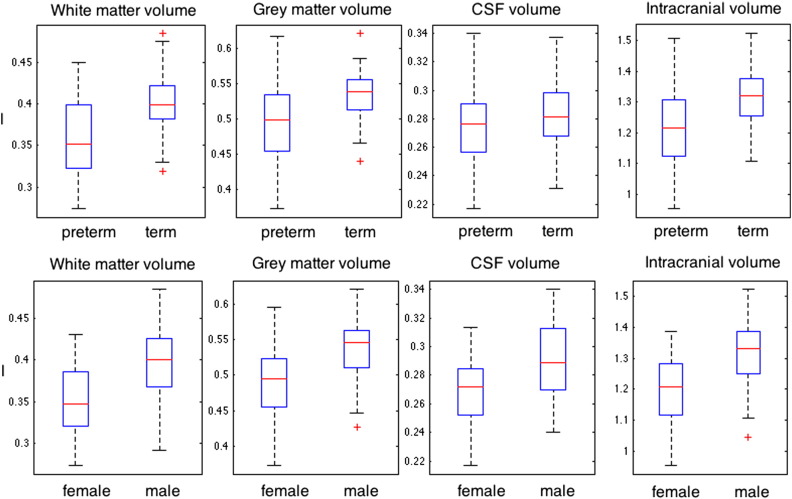
Tissue volume distributions (liters) for gray matter, white matter, CSF and intra-cranial volume grouped by top row: preterm against term volume and bottom row: male against female tissue volumes.

Fig. 1 shows a T2 map and three-class tissue segmentation for a single subject. The tissue volume results for all subjects analyzed are shown in Fig. 2; average total intracranial volume, gray matter volume, white matter volume and CSF volume are all lower in the extremely preterm group compared to controls (with p-values of 0.0018, 0.0143 and 0.0041 and 0.17 respectively). The bottom row of Fig. 4 shows significantly lower volume in females (0.35 ± 0.04 l for white matter) relative to males (0.40 ± 0.05 l for white matter) for all tissue types for white, gray, CSF and intracranial volume (p < 0.001).

**Fig. 3:**
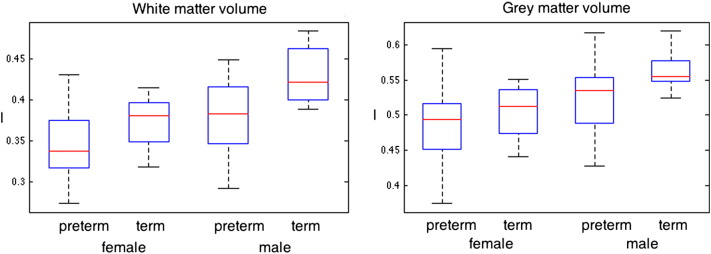
Tissue volume distributions for gray matter, white matter, CSF and intra-cranial volume grouped by preterm status and gender.

Fig. 3 shows the distribution of tissue volumes, separated by both preterm status and gender. Greater overall gray and white matter volumes are observed in the male group. Term born males have significantly larger white matter volume than preterm born males (p = 0.0125, 0.43 ± 0.04 l compared to 0.38 ± 0.04 l). Preterm born males have brain volumes comparable to term-born females (0.37 ± 0.03 l), whom in turn have larger brain volumes than preterm born females (0.35 ± 0.04 l) with p = 0.082. Within gender, the ratios of the average preterm/term-born brain volumes are similar at 0.93 for female and 0.90 for males.

### 3.2 T2 values in the preterm adolescent brain

**Fig. 4:**
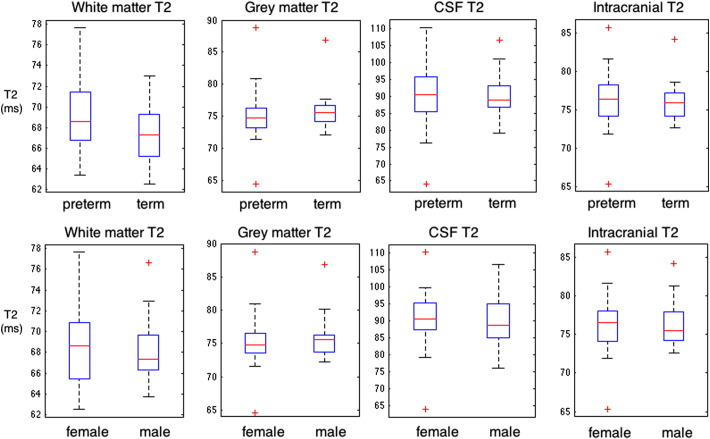
Tissue T2 distributions for gray matter, white matter, and CSF grouped by top row: preterm against term volume and bottom row: male against female T2 values.

Fig. 4 shows T2 distributions for groups separated by prematurity and gender. Significance is only reached between preterm and term white matter T2 in which the T2 of preterm white matter is slightly higher than that in term born white matter (p = 0.04, with T2 = 69.2 ± 3.6 in preterms compared to 67.3 ± 2.6 in term-borns). This difference is no longer significant when controlling for brain volume.

**Fig. 5:**
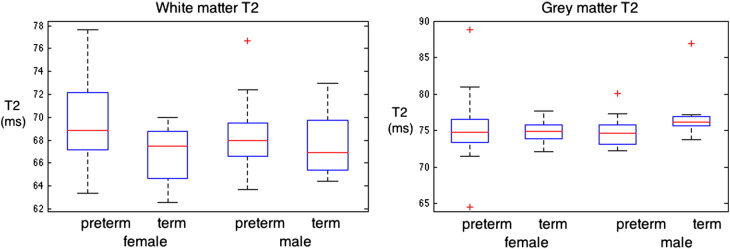
Tissue volume distributions (ms) for gray matter, white matter, CSF and intra-cranial volume grouped by preterm status and gender.

Fig. 5 shows the distribution of tissue T2 values, separated by both preterm status and gender. Significant differences in gray matter T2 are not observed between groups. The distribution of white matter T2 value in term born males is not significantly different from that in preterm born males (p = 0.58). The distribution of T2 values between term born and preterm born females does just reach significance (p = 0.039), with preterm-born females having on average a T2 that is 4% higher than their term-born peers (69.5 ± 3.8 ms compared to 66.8 ± 2.4 ms).

### 3.3 Multi-compartment myelin water fraction results

**Fig. 6:**
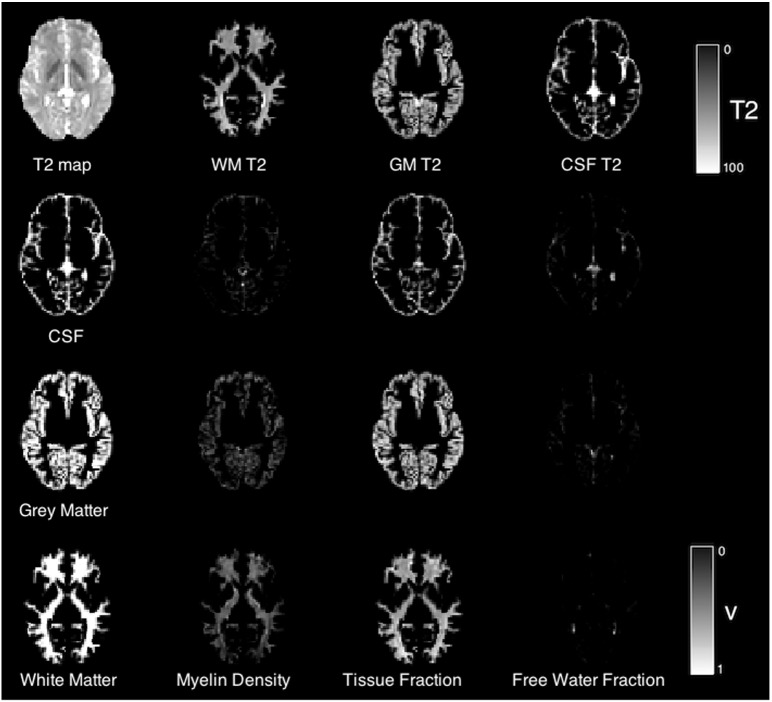
Example T2 map and tissue segmentation (top row) and subsequent rows: corresponding three-class tissue type segmentation overlaid on volume fraction maps for the three-component T2 model.

In the absence of significant differences in T2 value between preterm and term groups, we now investigate the tissue composition estimated by the three-compartment model. Fig. 6 illustrates, for one case, the spatial distribution of each volume fraction. The top row shows the T2 distribution overlaid with each layer of the segmentation. Subsequent rows show the distribution of each volume fraction with each layer of the segmentation. In white matter, a short T2 component is clearly identifiable, which extends more weakly into the gray matter. The tissue volume fraction has a strong component in each layer of the tissue segmentation, suggesting that there is an influence of partial voluming on the gray matter T2 estimates of the following section. In this section we focus on the white matter composition.

**Fig. 7:**
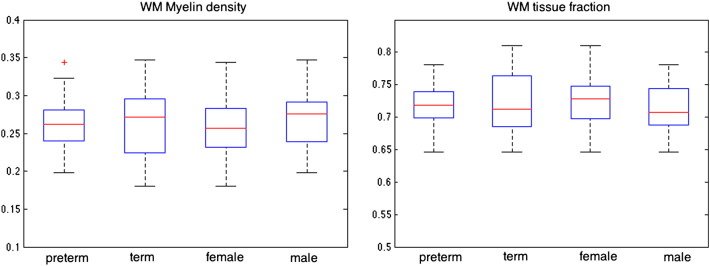
Multi-component tissue volume fractions for white matter myelin density and white matter tissue fraction grouped preterm against term volume and male against female.

**Fig. 8:**
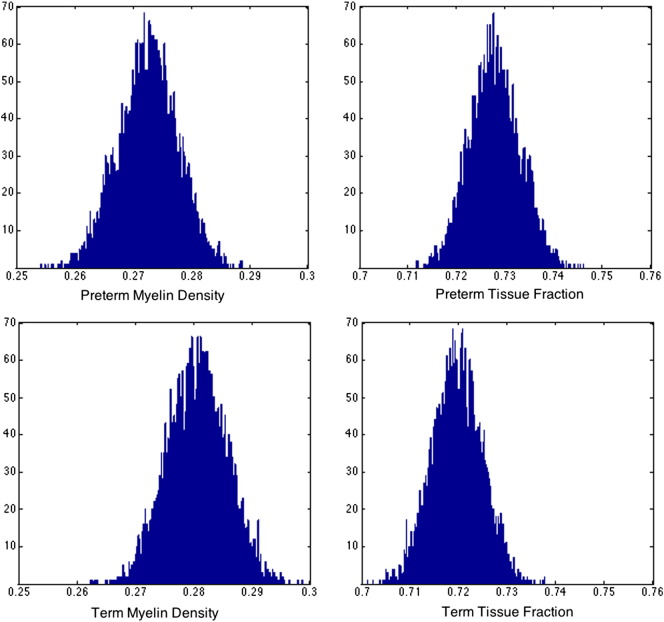
MCMC analysis of myelin density and tissue volume fraction precision for preterm (top row) and term (bottom row) groups.

Fig. 7 summarizes the previous group distributions of the myelin density and tissue fractions. WM myelin density was similar in EP and term subjects (0.25 ± 0.06 v 0.26 ± 0.05, respectively) and between males and females (0.26 ± 0.04 v 0.25 ± 0.06). Results of the MCMC analysis are shown in Fig. 8. The width of the distribution of mean possible parameter estimates for the myelin water fraction reinforces the results above that there is no detectable difference between preterm and term white matter myelin density. Assuming that these distributions are reasonably normal, preterm white matter density is 0.27 ± 0.005, compared to 0.28 ± 0.005 for the term group calculated for white matter signal averaged across all white matter voxels within each group.

## 4 Discussion

Our results show that tissue composition assessed using T2 relaxometry appears similar in extremely preterm compared to term born subjects but that total brain volume is reduced in the extremely-preterm cohort relative to their term-born peers. More specifically, tissue T2 values and myelin water fraction values are not significantly different between the two groups in either gray or white matter. In contrast though, brain volumes are significantly lower in the EP group and the size of the difference approximates to that between males and females. Although we observe a significant difference in average white matter T2 value between males and females, this difference is not significant after correction for brain tissue volume, which suggests that despite extreme prematurity, these individuals may have a normal appearing tissue composition, at least on T2 weighted imaging.

Previous work, especially work in neonates [5], [13], has shown that at term equivalent age, tissue properties are different between preterm born babies and their term-born peers. Specifically in preterm-born neonates the apparent white matter signal intensity on T2 weighted MRI appears higher, which is also associated with a higher single-component T2 value. Our work is the first to show that these differences might be mitigated in longer term development and that although tissue volume differences remain, tissue composition does not vary detectably on MRI.

One of the main strengths of this study is that it is one of the first to describe the appearance of the extremely preterm brain in adolescence and the study of multi-compartment T2 relaxometry we believe is currently unique. The acquisition of T2 relaxometry in large studies is relatively rare, particularly with multiple-echoes and thus we believe that our work is of wider importance. However, our study does have a number of limitations. It is worth noting that the individuals in this study were all able to consent to and tolerate the MR acquisition described in this work. This may imply that they are very able individuals and thus, differences in structure and function would be expected to be quite subtle. Furthermore the number of individuals is not large; comparing 46 EP adolescents with 20 adolescent controls. A larger cohort might cause some of the insignificant differences that we see in myelin water fraction to become significant.

Our future work will investigate the correlations of individual tissue composition and brain volume with functional outcome. This will allow us to specialize our image analysis paradigm to better characterize sub-cohorts of the EP group which is neuropsychologically diverse. This will be benefitted by the acquisition of a larger cohort and the associated increase in more specific definitions of function or disability type. The incorporation of additional MR contrast such as diffusion weighted imaging may help investigate additional structural differences in the brain tissue of these at risk adults.

In summary, research into the early adult preterm brain is an important area of research. The long term impact of extreme prematurity on quality of life is currently unknown and studies such as this, and neuroimaging studies in general, represent the best way to observe the neural substrates of functional deficit. Whether this specific study reflects evidence of a homogenisation in brain tissue composition at or before adolescence, or simply that the cohort is biased towards those subjects who are able to tolerate an MRI requires further investigation.
